# Impaired synaptosome phagocytosis in macrophages of individuals with autism spectrum disorder

**DOI:** 10.1038/s41380-025-03002-3

**Published:** 2025-04-04

**Authors:** Yuki Nishi, Michihiro Toritsuka, Ryohei Takada, Mitsuru Ishikawa, Rio Ishida, Yoshinori Kayashima, Takahira Yamauchi, Kazuki Okumura, Tsutomu Takeda, Kazuhiko Yamamuro, Minobu Ikehara, Yuki Noriyama, Kohei Kamikawa, Shuhei Murayama, Osamu Ichikawa, Hidetaka Nagata, Hideyuki Okano, Nakao Iwata, Manabu Makinodan

**Affiliations:** 1 Department of Psychiatry, Nara Medical University School of Medicine, Kashihara, Nara 634-8522 Japan; 2 Department of Psychiatry, Fujita Health University School of Medicine, Toyoake, Aichi 470-1192 Japan; 3 Division of Transformative Psychiatry and Synergistic Research, International Center for Brain Science (ICBS), Fujita Health University School of Medicine, Toyoake, Aichi 470-1192 Japan; 4 Department of Physiology, Keio University School of Medicine, Shinjuku-ku, Tokyo 160-8582 Japan; 5 Division of CNS Regeneration and Drug Discovery, International Center for Brain Science (ICBS), Fujita Health University School of Medicine, Toyoake, Aichi 470-1192 Japan; 6 Center for Health Control, Nara Medical University School of Medicine, Kashihara, Nara 634-8522 Japan; 7 Research and Development, Sumitomo Pharma Co., Ltd., Osaka city, Osaka 554-0022 Japan; 8 Keio University Regenerative Medicine Research Center Research Gate Building TONOMACHI 2-4F, 3-25-10 Tonomachi, Kawasaki-ku, Kawasaki, Kanagawa 210-0821 Japan

**Keywords:** Autism spectrum disorders, Neuroscience

## Abstract

Dendritic spine abnormalities are believed to be one of the critical etiologies of autism spectrum disorder (ASD). Over the past decade, the importance of microglia in brain development, particularly in synaptic elimination, has become evident. Thus, microglial abnormalities may lead to synaptic dysfunction, which may underlie the pathogenesis of ASD. Several human studies have demonstrated aberrant microglial activation in the brains of individuals with ASD, and studies in animal models of ASD have also shown a relationship between microglial dysfunction and synaptic abnormalities. However, there are very few methods available to directly assess whether phagocytosis by human microglia is abnormal. Microglia are tissue-resident macrophages with phenotypic similarities to monocyte-derived macrophages, both of which consistently exhibit pathological phenotypes in individuals with ASD. Therefore, in this study, we examined the phagocytosis capacity of human macrophages derived from peripheral blood monocytes. These macrophages were polarized into two types: those induced by granulocyte-macrophage colony-stimulating factor (GM-CSF MΦ, traditionally referred to as “M1 MΦ”) and those induced by macrophage colony-stimulating factor (M-CSF MΦ, traditionally referred to as “M2 MΦ”). Synaptosomes purified from human induced pluripotent stem cell-derived neuron were used to assess phagocytosis capacity. Our results revealed that M-CSF MΦ exhibited higher phagocytosis capacity compared to GM-CSF MΦ, whereas ASD-M-CSF MΦ showed a marked impairment in phagocytosis. Additionally, we found a positive correlation between phagocytosis capacity and *cluster of differentiation 209* expression. This research contributes to a deeper understanding of the pathobiology of ASD and offers new insights into potential therapeutic targets for the disorder.

## Introduction

Autism spectrum disorder (ASD) is a complex neurodevelopmental disorder characterized by deficits in social communication and interaction and restricted repetitive patterns of behavior and interests [1]. Although its etiology and pathogenesis have not yet been fully elucidated, dendritic spine abnormalities are believed to be one of the critical pathogeneses of ASD. Limited human induced pluripotent stem cell (hiPSC) models of ASD have been reported to have decreased spine density [2, 3], while, predominantly, postmortem brain studies of ASD have shown increased dendritic spine density [4–6]. For example, greater spine densities were observed in ASD subjects compared to control subjects, particularly in layer II pyramidal neurons in the frontal, temporal, and parietal lobes and layer V in the temporal lobe [7], and increased spine density was observed in the amygdala of young ASD brains [8]. In addition, longitudinal MRI studies have shown increased brain volume and cortical surface area in children with ASD before 2 years of age, which corresponds to the onset of social impairment characteristic of ASD [9]. Furthermore, human functional magnetic resonance imaging (MRI) studies have shown functional hyperconnectivity across multiple brain regions, including cortical and subcortical areas, in children with ASD [10]. According to the findings of these human imaging studies, an excess of spines may affect both brain structure and function.

Over the past decade, the importance of microglia in brain development has become increasingly apparent. Throughout human brain development, synapses increase rapidly from the perinatal to postnatal periods, peak during childhood, and gradually decline during adolescence and adulthood [11, 12]. During this process, excess synapses are eliminated while the appropriate synapses mature. It is worth noting that microglia play a crucial role in this process by pruning synapses through phagocytosis and releasing trophic factors [13–15]. Therefore, microglial abnormalities can result in synaptic dysfunctions, which may be the basis for the pathogenesis of ASD. A positron emission tomography study and a few postmortem studies have revealed aberrant microglial activation in the brains of individuals with ASD [16–18]. Several studies have identified synaptic and microglial abnormalities in mouse models of ASD. In mice lacking the *Fmr1* gene, which is associated with an increased susceptibility to ASD [19], researchers observed an increase in dendritic spine density but a decrease in mature dendritic spines in the adult hippocampus. Furthermore, microglia-mediated synaptic pruning was reduced in the CA1 region of Fmr1-knockout (KO) mice compared to wild type mice on postnatal day 21, which is the peak of synaptic pruning in the mouse hippocampus [20]. In a study using a maternal immune activation mouse model, which replicated maternal infection during pregnancy as a risk factor for ASD, the results indicated an increase in the number of spines in the hippocampus and a decrease in the expression of fractalkine microglial receptors (C-X3-C motif chemokine receptor 1; CX3CR1), a key factor in mediating the pruning process [21]. In fact, Cx3cr1-KO mice show impaired synaptic pruning, social interaction deficits, and increased repetitive behaviors characteristic of ASD [22]. Furthermore, it has been suggested that triggering receptor expressed on myeloid cells (TREM) 2 is associated with the microglia-mediated pruning process, and that Trem2-KO mice display increased synaptic density in the hippocampus and exhibited behavioral defects similar to those observed in individuals with ASD [23]. Additionally, the TREM2 protein is reduced in the postmortem brains of individuals with ASD as well as Nasu-Hakola disease [23, 24] and TREM2-KO hiPSC-derived microglia demonstrated decreased phagocytosis of synaptosomes [25]. Therefore, the relationship between microglial dysfunction and synaptic abnormalities in the mouse model of ASD is clear. However, there are very limited ways to directly measure whether phagocytosis by human microglia is abnormal.

Although the origin of microglia differs from that of macrophage, particularly monocyte-derived intravascular macrophages [26], microglia are still considered tissue-resident macrophages. Recent studies have shown that not only central nervous system, but also peripheral immune and inflammatory systems are involved in the onset of ASD through their effects on brain development [27]. Our mouse model studies indicated a correlation between the expression of neurotrophic factors in microglia and peripheral blood mononuclear cells (PBMCs) [28, 29]. These results suggests that it may be possible to evaluate the state of microglia to some extent by examining peripheral cells. Therefore, our group has conducted studies using human macrophages differentiated from peripheral blood monocytes and polarized into two types: granulocyte-macrophage colony-stimulating factor induced macrophages (GM-CSF MΦ; classic nomenclature “M1 MΦ”) and macrophage colony-stimulating factor induced macrophages (M-CSF MΦ; classic nomenclature “M2 MΦ”). The results showed that the expression of tumor necrosis factor-α (TNF-α), a pro-inflammatory cytokine, in GM-CSF MΦ was markedly higher in individuals with ASD than in typically developed (TD) individuals [30]. Furthermore, our other study using a co-culture system of hiPSC-derived neurons and macrophages showed that GM-CSF MΦ affects human neurons by inhibiting the outgrowth of microtubule-associated protein 2 (MAP2)-positive dendrites, and this inhibitory effect is more severe in GM-CSF MΦ of individuals with ASD than in TD individuals [31]. Although the diversity of microglia and macrophages cannot be solely explained by the polarized spectrum [13, 32–34], it is evident that a polarized phenotype is present in macrophages in vitro. Investigating the function of this phenotype may lead to novel discoveries about ASD.

In this study, we hypothesized that macrophages in individuals with ASD exhibit lower phagocytosis of synapses compared to those of TD individuals. To test this hypothesis, we added synaptosomes from hiPSC-derived neurons into the culture medium of macrophages and subsequently assessed their phagocytosis capacity and molecular mediators.

## Materials and methods

### Participants and clinical assessments

Twenty individuals with ASD (mean age: 29.0 ± 6.10 years, five females) and 18 TD individuals (mean age: 32.4 ± 5.23 years, four females) of Japanese ethnicity were enrolled. All participants were born and had been livinwith g in Japan. The participation criteria and the assessment of clinical features were the same as those described previously [30]. Briefly, individuals with ASD were recruited from the outpatient service of the Department of Psychiatry at Nara Medical University Hospital. The diagnosis of ASD was based on the Diagnostic and Statistical Manual of Mental Disorders, Fifth Edition criteria. At least two experienced psychiatrists examined each individual separately, and a diagnostic consensus was reached. A more detailed evaluation was performed by psychiatrists and trained staff using the Autism Diagnostic Observation Schedule-2 (ADOS-2) [35]. Autism symptom severity was assessed via self-reporting using the Autism Quotient-Japanese version (AQ-J) [36, 37]. All participants had an average intelligence of the full intelligence quotient (FIQ) of 70 or higher, as estimated using the Similarities and Symbol Search Subtests of the Wechsler Adult Intelligence Scale, 3^rd^ ed. [38]. Participants had no other neurological disorders, mental illnesses, infectious diseases, autoimmune diseases, or steroid use. This study was approved by the appropriate ethics committees of Nara Medical University. This study was conducted in accordance with the Ethics Code of the World Medical Association (Declaration of Helsinki) for experiments involving humans. All participants were given a complete description of the study and provided written informed consent prior to enrollment. The sample size was determined through calculation based on the effect size of 0.85 and the power of 0.8.

### Neuronal differentiation of hiPSCs

Human iPSC culture and neuronal differentiation were performed as the previously described method [31, 39]. The details are provided in Supplementary Methods. Briefly, two healthy control human iPSC lines, 201B7 [40] and 1008C15 [31, 41], were prepared and cultured in StemFit AK02N (Ajinomoto, Tokyo, Japan), seeded in a 35 mm culture dish coated with iMatrix-511 silk (Nippi, Tokyo, Japan) without feeder cells at 37 °C in humidified air containing 5% CO2. Then, the following plasmid vectors were co-transfected to establish *NEUROG2*-inducible hiPSCs: PB-TET-PH-lox66FRT-NEUROG2, pCMV-HyPBase-PGK-Puro, and PB-CAGrtTA3G-IH. To generate glutamatergic neurons, these *NEUROG2* -inducible hiPSCs were dissociated and seeded on poly-ornithine (Sigma-Aldrich) and iMatrix-511 silk-coated coverslips in 24-well plates at a density of 5 × 10^4^ cells/well and cultured in neural induction medium. After 5 days, the medium was replaced with a neuron culture medium. The medium was half-replaced twice a week for up to 56 days.

### Purification of synaptosomes from hiPSC-derived neurons and pHrodo-Red labeling

Synaptosomes were isolated from hiPSC-derived neurons using Syn-PER^TM^ Synaptic Protein Extraction Reagent (Thermo Fisher Scientific Inc.) according to the manufacturer’s protocol, with slight modifications. Briefly, after aspiration of culture medium, neurons were washed twice with ice-cold PBS, and 400 μl of Syn-PER^TM^ Reagent supplemented with protease and phosphatase inhibitor (Thermo Fisher Scientific Inc.) was added to neurons cultured in a 35 mm dish. The neurons were then collected from the plate using a cell scraper to transfer them to a microcentrifuge tube and centrifuged at 1200×g for 10 min at 4 °C. The supernatant was subsequently transferred to a new tube and centrifuged at 15000×g for 20 min at 4 °C. The supernatant was removed, and the synaptosome pellet was resuspended in the appropriate amount of Syn-PER^TM^ Reagent and stored in 5% DMSO at −80 °C for phagocytosis assay and western blotting. The residual neuronal pellet was lysed by RIPA buffer supplemented with protease and phosphatase inhibitor and stored at −80 °C for western blotting. Total protein concentration was determined using a Pierce^TM^ BCA protein assay kit (Thermo Fisher Scientific Inc.).

For labeling synaptosomes, pHrodo^TM^ Red succinimidyl ester (Thermo Fisher Scientific Inc.) was added to the dissolved synaptosomes and the mixture was incubated for 30 minutes at room temperature in the dark. This pH-sensitive dye binds non-specifically to proteins and increases its fluorescence as the pH levels of its surroundings become more acidic in the post-phagocytic phagolysosomal compartments of cells.

### Monocyte isolation and macrophage differentiation

Monocyte were isolated using a magnetic-activated cell sorting system (Miltenyi Biotec, Bergisch Gladbach, Germany) and a Human M1 or M2 Macrophage Differentiation Kit (R&D Systems), according to the manufacturer’s protocol and a previously described method [30, 31]. The details are provided in Supplementary Methods. Briefly, whole human blood samples were obtained by venipuncture. The PBMCs were immediately isolated using density-gradient centrifugation. Cluster of differentiation (CD) 14+ monocytes were isolated from PBMCs using a magnetic-activated cell sorting system with CD14 microbeads (Miltenyi Biotec). For macrophage differentiation, CD14+ monocytes were seeded in 12-well plates coated with poly-L-lysine (IWAKI, Shizuoka, Japan) at a density of 1 × 10^6^ cells/ml, and then cultured in serum-free medium containing recombinant human GM-CSF or M-CSF, respectively. GM-CSF MΦ or M-CSF MΦ were collected on day 6 for the phagocytosis assay and qRT-PCR analysis.

### Phagocytosis assays of synaptosomes

Some of the collected macrophages were seeded in a 35 mm glass-bottom cell culture dish with four compartments (Greiner Bio-One) in a neuron culture medium. Two compartments were allocated for each macrophage cell type at a density of 1 × 10^5^ cells per compartment. Macrophages were incubated for 1 h at 37 °C in humidified air containing 5% CO2.

The pHrodo^TM^ Red-labeled synaptosomes were suspended in a neuron culture medium with a concentration of 12.5 μg/ml, and 5 μg of labeled synaptosomes were added to the cultured macrophages in each compartment. Immediately after the addition of labeled synaptosomes, macrophages were incubated at 37 °C in humidified air containing 5% CO2 using a Nikon Biostation immunofluorescence microscope (Nikon Instruments Inc., Tokyo, Japan). Four points were randomly selected from each compartment and time-lapse images were captured every 9 min for 90 min using a ×20 objective lens. At each capture point, the difference in fluorescence intensity of all macrophages immediately and 90 min after the addition of labeled synaptosomes was analyzed using NIH Image J software. The fluorescence intensity ratio was calculated to quantify phagocytosis capacity. Blinding was applied during data collection and analysis of individuals and macrophages subtypes.

### Knockdown of CD209 expression in M-CSF induced macrophages by siRNA

For siRNA gene silencing in M-CSF MΦ, we used the forward transfection method with minor modifications as previously described [42, 43]. Briefly, differentiating M-CSF MΦ adhered to the bottom of the 12-well plate on day 3 of culture were gently washed twice with warmed M-CSF MΦ medium, and 1333.3 μl of medium per well was added. The transfection mixture for each well, comprised of 586.7 µl warm medium, 60 µl HiPerFect transfection reagent (Qiagen, Hilden, Germany) and 20 µl ON-TARGETplus SMARTpool siRNA targeting CD209 (DC-SIGN) or non-targeting siRNA (Dharmacon, Thermo Fisher Scientific Inc.), was incubated at room temperature for 15−20 min, with gentle inversion of the mixture several times. The lipid-siRNA complexes were then added drop-wise onto the medium of adherent macrophages, and the plates were placed at 37 °C and 5% CO2. After 6 h, macrophage culture medium (2666.7 µl) with twice the concentration of M-CSF was added per well, and the plates were placed at 37 °C and 5% CO2 again. Differentiated macrophages were collected on day six using cell scrapers for phagocytosis assays and qRT-PCR.

### Quantitative reverse transcription-PCR

Total RNA was extracted from cultured cells using the AllPrep DNA/RNA/Protein Mini Kit (Qiagen), according to the manufacturer’s protocol. RNA concentration was determined by measuring absorbance at 260 nm. First-strand cDNA was synthesized from total RNA using an iScript kit (Bio-Rad Laboratories, Hercules, CA, USA) and quantitative RT-PCR was performed using SYBR Premix Ex TaqII (Tli RNaseH Plus, Takara Bio Inc., Shiga, Japan) with a QuantStudio 6 real-time PCR system (Applied Biosystems, Thermo Fisher Scientific Inc.). Relative quantification of target gene expression levels was performed following the delta CT method, using two constitutively expressed genes as internal controls: *β-actin* (*ACTB*) and *Cyclophilin A* (*CyA*). Primer sequences are shown in the Supplementary Methods.

### Statistical analysis

Differences in demographic characteristics (age, educational level, AQ total score, and estimated FIQ) between individuals with ASD and TD individuals were examined using an unpaired t-test after determined equal variances using the F test, and Fisher’s exact test was used to examine differences in the sex ratio. Comparisons of phagocytosis capacity and gene expression in GM-CSF MΦ and M-CSF MΦ between the TD and ASD groups were performed by two-way analysis of variance (ANOVA) with posthoc multiple comparison test using Tukey’s honest significant test (HSD). Data from the knockdown analysis were analyzes using repeated measures ANOVA with a posthoc multiple comparison test using the Dunnett’s multiple comparison test.

Simple linear regression analysis was used to examine the association between phagocytic capacity and *CD209* gene expression of M-CSF MΦ.

All statistical analyses were performed using Prism 10 software (GraphPad, Inc., La Jolla, CA, USA). Differences were considered statistically significant at p < 0.05. For the qRT-PCR analysis of six pattern recognition receptor genes, the Bonferroni correction was applied, and the threshold for statistical significance was set at p < 0.0083.

## Results

Demographic data for participants are presented in Table 1. There were no significant differences in age (t = 1.89, p = 0.0674), sex (p > 0.9999), FIQ score (t = 0.144, p = 0.886), or education (t = 1.31, p = 0.198) between the ASD and TD groups. All participants had higher FIQ scores than 80, and there was no significant difference in the variance of FIQ score between the two groups (F test, F = 2.354, p = 0.0818), thus there was no possibility that intellectual disability affected the results. As expected, individuals with ASD had significantly higher AQ-J scores than those with TD individuals (t = 7.53, p < 0.0001).

**Table 1:** Basic characteristics in each group.

	TD	ASD	
	(n = 18)	(n = 20)	
age, mean (SD)	32.4 (5.23)	29.0 (6.10)	t = 1.89, p = 0.0674
sex, male, n (%)	14 (78%)	15 (75%)	p > 0.9999*
estimated Full IQ, mean (SD)	108.5 (8.85)	107.9 (13.6)	t = 0.144, p = 0.886
education, year (SD)	16.8 (2.68)	15.8 (2.17)	t = 1.31, p = 0.198
AQ total score, mean (SD)	16.0 (5.83)	31.3 (6.58)	**t** = **7.53, p** < **0.0001**

unpaired t-test.

^*^Fisher’s exact test.

*ASD* autism spectrum disorder, *TD* typical development, *AQ* Autism Quotient, *SD* standard deviation, *IQ* intelligence quotient.

**Fig. 1:**
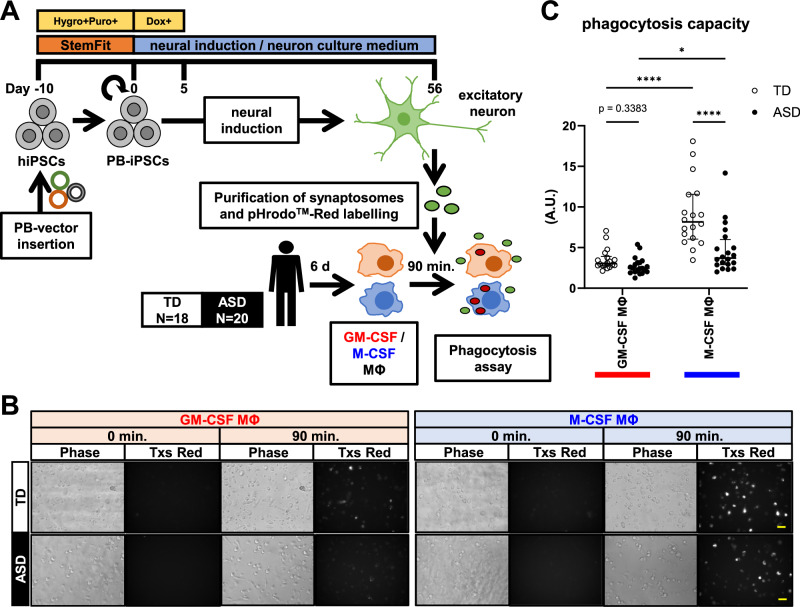
Phagocytosis assay of synaptosomes purified from hiPSC-derived neurons by macrophages. **A** Summary of the phagocytosis assay. Two hiPSC lines from healthy controls are differentiated into excitatory neurons and collected for synaptosome purification. **B** Representative images of phagocytosis of synaptosomes by GM-CSF MΦ and M-CSF MΦ of TD12 and ASD21. Images captured at the outset of the experiment and 90 min later in the phase contrast and Texas Red Filter, respectively, are presented. Scale bar: 30 μm. **C** Results of the phagocytosis assay. Phagocytosis capacity is significantly higher in M-CSF MΦ compared to GM-CSF MΦ. Furthermore, the phagocytosis capacity of ASD-M-CSF MΦ is significantly lower than that of TD-M-CSF MΦ. Two-way ANOVA test, F(1, 72) = 8.518, p = 0.0047 (interaction), F(1, 72) = 39.45, p < 0.0001 (MΦ polarity), F(1, 72) = 18.33, p < 0.0001 (group), with post hoc Tukey’s multiple comparison test. n (TD) = 18 and n (ASD) = 20. *p < 0.05, **p < 0.01, ***p < 0.001, ****p < 0.0001.

The protocol for neuronal differentiation, purification of synaptosomes and the synaptosome phagocytosis assay using macrophages is shown in Fig. 1A. For neuronal differentiation, we prepared two hiPSC lines from healthy controls. A previous study [39] and our earlier work [31] demonstrated that neurons derived from hiPSCs using the same induction method as in this study were predominantly glutamatergic excitatory neurons. Additionally, we showed neuronal viability and functional excitatory synaptic connectivity at DIV28 using the whole-cell patch-clamp technique [31]. The accuracy of synaptosome purification was validated by western blot analysis. The nuclear protein histone deacetylase 2 (HDAC-2) was efficiently excluded, while the presynaptic protein synaptophysin (SYP) and the postsynaptic protein postsynaptic density 95 (PSD95) and glutamate ionotropic receptor N-methyl-D-aspartate (NMDA) type subunit 2B (NR2B) were enriched in the synaptosomal fraction (Supplementary Fig. 1A, B).

### ASD-M-CSF MΦ have a lower phagocytosis capacity than TD-M-CSF MΦ

Before evaluating phagocytosis, we confirmed the polarization of differentiated macrophages by qRT-PCR on day 6. GM-CSF MΦ showed an inflammatory profile of high expression of *interleukin-1α (IL-1α)* and low expression of *IL-10*, while M-CSF MΦ showed an anti-inflammatory profile with an opposite expression pattern in both the TD and ASD groups (Supplementary Fig. 1C, D). The gene expression of *IL-1α* in GM-CSF MΦ was found to be significantly higher in the ASD group, which replicated our previous findings [31] with an increased sample size. To assess the phagocytosis of synaptosomes, macrophages were incubated with pHrodo^TM^ Red-labeled synaptosomes for 90 min. After phagocytosis, the fluorescence intensity increased due to the pH change inside the macrophages, and the fluorescence change was detected by time-lapse fluorescence microscopy (Fig. 1B, Supplementary Movies 1−8). Phagocytosis capacity was defined as the ratio of fluorescence intensity immediately after synaptosome addition to that 90 min later. The results showed that the phagocytosis capacity of M-CSF MΦ was significantly higher than that of GM-CSF MΦ in the TD and ASD groups; however, the difference was less pronounced in the ASD group than in the TD group. Additionally, phagocytosis capacity of M-CSF MΦ was generally lower in the ASD group than in the TD group (Fig. 1C). The observed differences in phagocytosis capacity may be attributed to the differences in cell maturity; however, the maturation speed of M-CSF MΦ was indistinguishable between the TD and ASD groups, as shown by the equivalent gene expression patterns of *CD68*, a glycosylated transmembrane protein localizing to plasma membrane as well as lysosomes and endosomes in mature macrophages, throughout the in vitro differentiation period (Supplementary Fig. 1E).

### ASD-M-CSF MΦ have lower expression of CD209 compared to TD-M-CSF MΦ

**Fig. 2:**
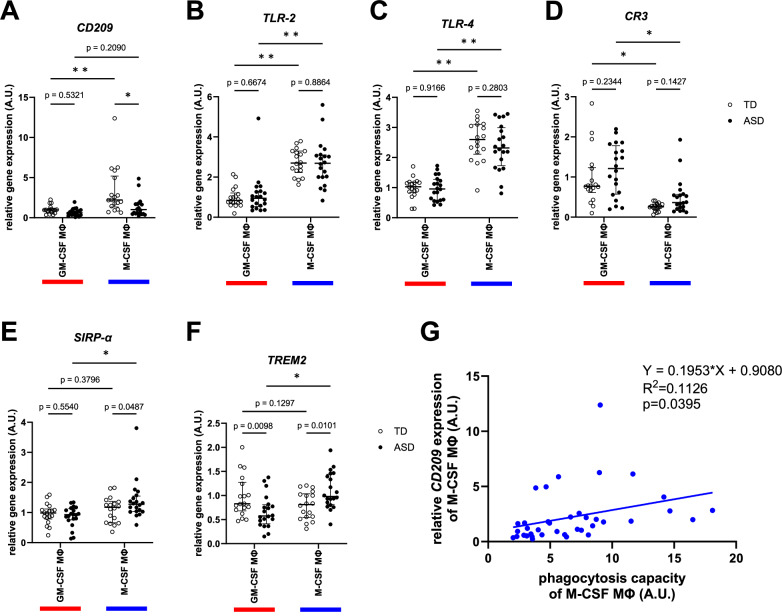
Gene expression analysis of phagocytosis-related receptors. **A–F** Gene expression analysis using qRT-PCR. Only *CD209* expression showed a pattern consistent with the phagocytosis capacity results. **A** Two-way ANOVA test, F(1, 72) = 4.841, p = 0.0310 (interaction), F(1, 72) = 15.57, p = 0.0002 (MΦ polarity), F(1, 72) = 9.536, p = 0.0029 (group), with post hoc Tukey’s multiple comparison test. **B** Two-way ANOVA test, F(1, 72) = 0.1652, p = 0.6856 (interaction), F(1, 72) = 68.51, p < 0.0001 (MΦ polarity), F(1, 72) = 0.04149, p = 0.8392 (group), with post hoc Tukey’s multiple comparison test. **C** Two-way ANOVA test, F(1, 72) = 0.4829, p = 0.4893 (interaction), F(1, 72) = 123.4, p < 0.0001 (MΦ polarity), F(1, 72) = 0.7115, p = 0.4017 (group), with post hoc Tukey’s multiple comparison test. **D** Two-way ANOVA test, F(1, 72) = 0.04004, p = 0.8420 (interaction), F(1, 72) = 32.16, p < 0.0001 (MΦ polarity), F(1, 72) = 3.595, p = 0.0620 (group), with post hoc Tukey’s multiple comparison test. **E** Two-way ANOVA test, F(1, 72) = 3.380, p = 0.0701 (interaction), F(1, 72) = 9.742, p = 0.0026 (MΦ polarity), F(1, 72) = 0.9949, p = 0.3219 (group), with post hoc Tukey’s multiple comparison test. **F** Two-way ANOVA test, F(1, 72) = 14.02, p = 0.0004 (interaction), F(1, 72) = 2.310, p = 0.1329 (MΦ polarity), F(1, 72) = 0.00005556, p = 0.9941 (group), with post hoc Tukey’s multiple comparison test. n (TD) = 18 and n (ASD) = 20. *p < 0.001, **p < 0.0001. **G** A simple linear regression analysis of phagocytosis capacity and *CD209* expression of M-CSF MΦ indicated a constant correlation (Y = 0.1953X + 0.9080, R2 = 0.1126, p = 0.0395).

Next, to uncover the molecular mechanisms behind the lower phagocytosis capacity observed in ASD-M-CSF MΦ, we performed qRT-PCR analysis in macrophages, focusing on cell surface receptors that are considered necessary for the recognition of phagocytic targets. This analysis included the pattern recognition receptors CD209 [44], TLR2, and TLR4 [45, 46], which regulate the phagocytosis of *E. coli* and beads in macrophages, as well as CR3 [47], TREM2 [23], and SIRP-α [48], which are associated with synapse pruning in microglia. Among these receptors, only *CD209* expression showed a consistent pattern with the results of phagocytosis capacity, as shown in Fig. 1C, significantly higher in M-CSF MΦ compared to GM-CSF MΦ and lower in ASD-M-CSF MΦ compared to TD-M-CSF MΦ (Fig. 2A−F). These results indicated that CD209 may play a significant role in synaptic phagocytosis. A correlation between phagocytosis capacity and *CD209* expression in M-CSF MΦ will support this hypothesis, as shown by simple linear regression analysis (Fig. 2G).

### Knockdown of CD209 expression by siRNA reduced phagocytosis capacity in TD-M-CSF MΦ

**Fig. 3:**
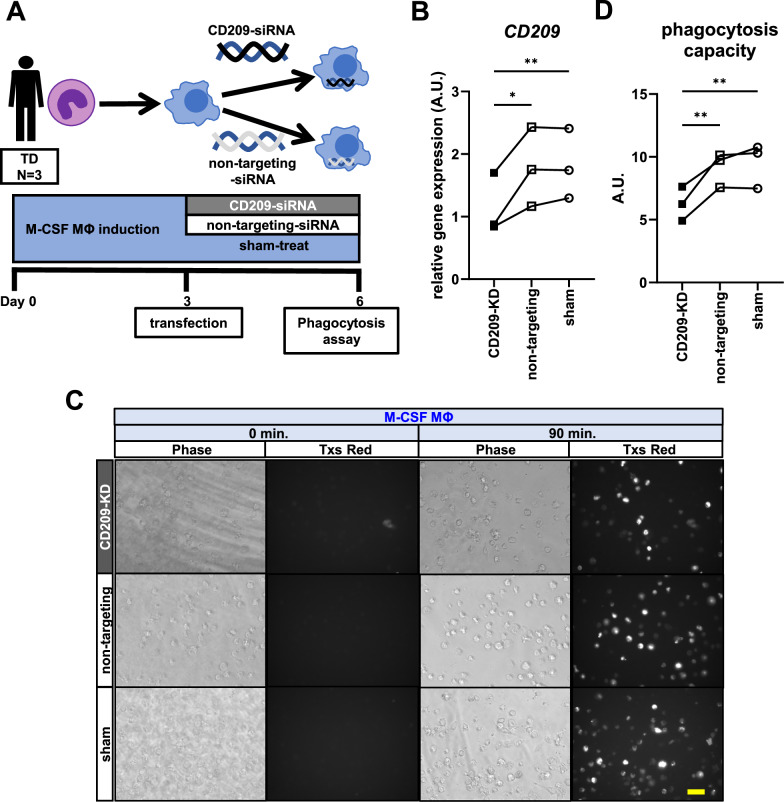
Knockdown experiment of CD209 in TD-M-CSF MΦ. **A** Summary of the knockdown experiment by siRNA in M-CSF MΦ from three TD individuals. **B** Gene expression of CD209 is significantly reduced in CD209-siRNA-transfected M-CSF MΦ compared to non-targeting-siRNA-transfected M-CSF MΦ and sham-treated M-CSF MΦ. Repeated measures ANOVA test, F(2, 4) = 19.86, p = 0.0084, with post hoc Dunnett’s multiple comparison test. **C** Representative images of phagocytosis by CD209-siRNA-transfected, non-targeting-siRNA-transfected, and sham-treated M-CSF MΦ of TD10, respectively. Images captured at the outset of the experiment and 90 min later in the phase contrast and Texas Red Filter, respectively, are presented. Scale bar: 30 μm. **D** Results of the phagocytosis assay. Phagocytosis capacity is significantly lower in CD209-siRNA-transfected M-CSF MΦ compared to non-targeting-siRNA-transfected M-CSF MΦ and sham-treated M-CSF MΦ. Repeated measures ANOVA test, F(2, 4) = 32.92, p = 0.0033, with post hoc Dunnett’s multiple comparison test.

To confirm the effect of CD209 on macrophage phagocytosis, we performed knockdown experiments using siRNA transfection. This protocol is illustrated in Fig. 3A. Briefly, differentiating M-CSF MΦ from three TD individuals were transfected with siRNA on day 3, and differentiated macrophages were collected on day 6 for the qRT-PCR and phagocytosis assay. First, we measured the gene expression of *IL-1α* and *IL-10*, and confirmed that macrophages maintained their polarity as M-CSF MΦ after transfection with siRNA or sham treatment, with comparable expression levels of these genes (Supplementary Fig. 2A, B). The expression of *CD209* was then analyzed. Transfection of siRNA targeting *CD209* into TD-M-CSF MΦ successfully decreased *CD209* expression as compared to that of non-targeting-siRNA-transfected MΦ and sham-treated MΦ (Fig. 3B). The phagocytosis assay was performed in these siRNA-transfected MΦ and sham-treated MΦ in the same manner as Fig. 1. As expected, phagocytosis capacity was significantly lower in CD209-siRNA-transfected MΦ compared to non-targeting-siRNA-transfected MΦ and sham-treated MΦ (Fig. 3C, D, Supplementary Movies 9−14). These findings indicate that CD209 is a crucial factor in macrophage phagocytosis.

To ascertain the mechanisms underlying the observed difference in *CD209* gene expression, we conducted a Single-Cell Assay for Transposase-Accessible Chromatin sequencing analysis (scATAC-seq). PBMCs from TD and ASD individuals matched for age and sex were used (Supplementary Table 1), and 500 monocytes from each individual were randomly sampled and analyzed. The results identified four ATAC-seq peaks around the CD209 gene region, although no significant difference was observed between TD and ASD groups (Supplementary Fig. 3A and B).

## Discussion

In this study, we assessed phagocytosis of synaptosomes in macrophages and found that M-CSF MΦ exhibited higher phagocytosis capacity compared to GM-CSF MΦ. Notably, this study is the first to reveal lower phagocytosis capacity of synaptosomes in ASD-M-CSF MΦ compared to TD-M-CSF MΦ, with a correlation to *CD209* gene expression. Although abnormalities in macrophages do not necessarily indicate similar issue in microglia [13], our findings support the hypothesis of abnormal synapse pruning in ASD pathogenesis and suggest that the same pathology may be present in macrophages as in microglia.

Microglia and macrophages exhibit a spectrum of phenotypes [13, 49]. While we identified functional abnormalities in M-CSF MΦ, other studies have reported a lower prevalence of peripheral blood “M2” macrophages in individuals with ASD [50], indicating potential issues with the M-CSF induced (“M2”) phenotype of macrophage/microglia in ASD.

Several studies, including animal models, neuroimaging, postmortem, and genetic and transcriptomic investigations, have suggested that abnormal microglial activation and synaptic dysfunction are crucial mechanisms in the ASD pathology. However, it is important to note that no direct evidence has yet been found for glia-mediated synaptic abnormalities in human ASD, and microglia may simply be responding to the surrounding neuronal pathology [15]. To address these issues, an in vitro model using patient-derived cells, as shown in this study, can be useful. Recently, methods for inducing microglia-like cells from peripheral blood monocytes [51], or iPS cells [52–54] have been established, allowing for the analysis of patient-derived cells with or without neurons. In neuropsychiatric disorders, a schizophrenia model using a co-culture of iPSC-derived neurons and induced microglia-like (iMG) cells showed higher phagocytosis of patient-derived iMG cells compared to healthy controls [55]. This finding aligns with the hypothesis that excessive synaptic pruning by microglia contributes to the reduced synaptic density observed in postmortem studies of schizophrenia [4], highlighting the utility of in vitro models. In comparison to the in vitro models utilizing microglia-like cells described above, our macrophage model was limited in that it only allows the indirect examination of microglia function. However, the significant advantage of the current method using macrophages is that testing can be completed in a short time (within 6 days) from peripheral blood collection [30, 31].

CD209, also known as a dendritic cell-specific inter-cellular adhesion molecular-grabbing nonintegrin (DC-SIGN), is a member of C-type lectin receptors that recognize specific carbohydrate structures of a wide range of pathogens, facilitating phagocytosis and antigen presentation to T cells [56–58]. It also recognizes self-glycoproteins to allow tolerance to self-antigens and mediates cellular processes such as cell signaling, cell adhesion, and migration [57, 59]. CD209 is primarily expressed in dendritic cells, tissue-resident macrophages, and PBMCs [60, 61]. Additionally, monocyte-derived macrophages cultured with IL-13 and microglia isolated from human brain tissue treated with M-CSF, IL-4, and IL-13 have been reported to express CD209 [62, 63]. In particular, these microglia are designated as “M2” phenotype cells, akin to macrophages in our present study. While CD209 is evidently involved in the pathogen phagocytosis by dendritic cells and macrophages [44, 64–69], its role in synapse pruning by microglia remains unclear. To our knowledge, there is only one report demonstrating the relationship between CD209 expression and high synaptosome phagocytosis in microglial cells (iTF-Microglia), in which the overexpression of CD209 increased the phagocytosis of both beads and synaptosomes, but had a smaller effect on bead phagocytosis [53]. Our study is the second to show a correlation between *CD209* expression and synaptosome phagocytosis and the first to suggest that this process may contribute to the pathology of ASD. Our scATAC-seq revealed no epigenetic differences in monocytes between the TD and ASD groups, indicating that the chromatin structures relevant to *CD209* were identical at least at this stage. Notably, *IL-1α* expression was higher in ASD-GM-CSF MΦ compared to TD-GM-CSF MΦ, which could influence *CD209* expression. However, given that there was no correlation between *IL-1α* expression in ASD-GM-CSF MΦ and *CD209* expression in ASD-M-CSF MΦ (Supplementary Fig. 2C), and that the knockdown of *CD209* did not affect *IL-1α* expression in the knockdown experiment, it is unlikely that *CD209* expression was affected by *IL-1α*. Future studies are needed to elucidate the function and mechanisms of synaptic pruning, these findings provide new insights into therapeutic targets for ASD.

One limitation of our present study is the small sample size, restricted to the adult Japanese population, making it unclear whether the results are applicable to a more diverse general population. To investigate a potential association between phagocytosis capacity and the demographic characteristics of individuals with ASD, we performed multiple linear regression analyses. However, no statistically significant associations were observed (Supplementary table 2). This suggests that a larger sample size may be necessary to identify any meaningful correlations. Additionally, there is no direct evidence linking macrophage phenotypes with microglia. Phagocytosis capacity was assessed using synaptosomes obtained only from excitatory neurons, leaving it unknown whether abnormal phagocytosis persists in the in vivo brain environment with GABAergic inhibitory neurons, astrocytes, and other cells. Furthermore, the neurons were derived from two healthy control subjects, preventing analysis of neuron-macrophage interactions in individuals with ASD. In addition, as the synaptosomes were derived from different individuals than those from whom the macrophages were collected, it is possible that allogenic immune responses to non-self components may have influenced the phagocytosis capacity. As the pHrodo^TM^ dyes enable the detection of both endocytosis and phagocytosis, we were unable to clearly distinguish between these processes. Additionally, we performed an overexpression experiment to determine whether the overexpression of *CD209* could restore the phagocytosis capacity of macrophages in individuals with ASD. However, the level of gene expression varied among individuals, and changes in phagocytosis capacity exhibited an individual-specific pattern (Supplementary Fig. 4). As a result, we were unable to determine the optimal level of *CD209* expression required for effective phagocytosis. Lastly, we cannot rule out the involvement of factors other than CD209 or their interactions with CD209. Nonetheless, using simplified culture systems, we identified macrophage abnormalities in individuals with ASD.

In conclusion, we demonstrated that ASD-M-CSF MΦ exhibits lower synaptosome phagocytosis capacity compared to TD-M-CSF MΦ, and that CD209 is associated with this phagocytosis capacity of M-CSF MΦ. These results are useful to understand the pathobiology for ASD and for future drug discovery.

## Supplementary information

Supplemental Information

Supplementary Figure 1

Supplementary Figure 2

Supplementary Figure 3

Supplementary Figure 4

Supplementary Table 1

Supplementary Table 2

Supplementary Movie 1

Supplementary Movie 2

Supplementary Movie 3

Supplementary Movie 4

Supplementary Movie 5

Supplementary Movie 6

Supplementary Movie 7

Supplementary Movie 8

Supplementary Movie 9

Supplementary Movie 10

Supplementary Movie 11

Supplementary Movie 12

Supplementary Movie 13

Supplementary Movie 14

## Data Availability

The datasets used and/or analyzed during the current study are available from the corresponding author on reasonable request.
