# Supplementary material for: Impaired synaptosome phagocytosis in macrophages of individuals with autism spectrum disorder
Source: Mol Psychiatry. 2025 Apr 4;30(8):3837–45. doi: 10.1038/s41380-025-03002-3 (PMC12240830; doi:10.1038/s41380-025-03002-3)
Supplement: Supplementary file 1 — Supplemental Information [file 41380_2025_3002_MOESM1_ESM.docx]

**Supplemental Information**

**Supplementary Methods**

**Neuronal differentiation of hiPSCs**

Two healthy control human iPSC lines were cultured in StemFit AK02N (Ajinomoto, Tokyo, Japan), seeded in a 35 mm culture dish coated with iMatrix-511 silk (Nippi, Tokyo, Japan) without feeder cells at 37°C in humidified air containing 5% CO2. The following plasmid vectors were used to establish *NEUROG2* -inducible hiPSCs: PB-TET-PH-lox66FRT-NEUROG2, pCMV-HyPBase-PGK-Puro, and PB-CAGrtTA3G-IH. These vectors were co-transfected into hiPSCs using Gene Juice Transfection Reagent (Novagen, Madison, Wisconsin, USA). The transfectants were cultured in StemFit AK02N supplemented with 150 μg/ml hygromycin (Wako Pure Chemical Industries, Ltd., Osaka, Japan) and 0.1−1.0 μg/ml puromycin (Sigma-Aldrich, St. Louis, MO, USA). To generate glutamatergic neurons, these *NEUROG2* -inducible hiPSCs were dissociated and seeded on poly-ornithine (Sigma-Aldrich) and iMatrix-511 silk-coated coverslips in 24-well plates at a density of 5× 10^4^ cells/well and cultured in neural induction medium; Neurobasal Plus medium supplemented with B-27, 1× Glutamax (Invitrogen, Thermo Fisher Scientific Inc., Waltham, MA, USA), 20 μM Y-27632 (Wako Pure Chemical Industries, Ltd.), 20 μM DAPT (Sigma-Aldrich), 100 μg/ml G418 (Nacalai Tesque, Kyoto, Japan) and 1 μg/ml doxycycline (Wako Pure Chemical Industries, Ltd.). After five days, the medium was replaced with neuron culture medium: Neurobasal Plus medium supplemented with B-27, 1× Glutamax, 10 ng/ml brain-derived neurotrophic factor (BDNF; R&D Systems, Minneapolis, MN, USA), 10 ng/ml glial cell line-derived neurotrophic factor (GDNF; Almone-Labs, Jerusalem, Israel), 200 μM L-ascorbic acid (Sigma-Aldrich), 200 μM dibutyryl cyclic adenosine monophosphate sodium salt (Nacalai Tesque), 10 ng/ml neurotrophin-3 (Almone-Labs), 1× CultureOne Supplement (GIBCO, Thermo Fisher Scientific Inc.) and 1% penicillin-streptomycin mixed solution (Nacalai Tesque). The medium was half-replaced twice a week for up to 56 days.

**Monocyte isolation and macrophage differentiation**

Monocyte isolation was performed using a magnetic-activated cell sorting system (Miltenyi Biotec, Bergisch Gladbach, Germany) and a Human M1 or M2 Macrophage Differentiation Kit (R&D Systems), according to the manufacturer’s protocol. Briefly, whole-human blood samples were obtained by venipuncture from all participants. PBMCs were immediately isolated by density-gradient centrifugation using the separation medium Lymphoprep (Axis-Shield, Oslo, Norway) and Leucosep separation tubes (Greiner Bio-One, Kremsmünster, Austria). Subsequently, CD14+ monocytes were isolated from the PBMCs using a magnetic-activated cell sorting system with CD14 microbeads (Miltenyi Biotec). For macrophage differentiation, CD14+ monocytes were resuspended in a phosphate-buffered saline (Wako Pure Chemical Industries, Ltd.) containing 0.5% bovine serum albumin (Sigma-Aldrich), 2 mM ethylenediaminetetraacetic acid and 1% penicillin-streptomycin mixed solution (Nacalai Tesque). Cells were seeded in 12-well plates coated with poly-L-lysine (IWAKI, Shizuoka, Japan) at a density of 1× 10^6^ cells/ml and then cultured in serum-free base medium containing recombinant human GM-CSF or recombinant human M-CSF, respectively, at 37°C in humidified air containing 5% CO2. On day 3, half of the culture medium was replaced with fresh medium, and GM-CSF MΦ or M-CSF MΦ were collected on day 6 using a cell scraper for phagocytosis assay and qRT-PCR analysis.

**Quantitative reverse transcription-PCR**

The primer sequences were as follows: *β-actin*, forward 5´- GATGTGGATCAGCAAGCA-3´, reverse 5´-AGAAAGGGTGTAACGCAACTA-3´; *CyA*, forward 5´-GCAGACAAGGTCCCAAAG-3´, reverse 5´-GAAGTCACCACCCTGACAC-3´; *IL-1α*, forward 5´-TGTATGTGACTGCCCAAGATGAAG-3´, reverse 5´-AGAGGAGGTTGGTCTCACTACC-3´; *IL-10*, forward 5´-GCCTAACATGCTTCGAGATC-3´, reverse 5´-TGATGTCTGGGTCTTGGTTC-3´;　*TNF-α*, forward 5´-GGCAGTCAGATCATCTTCTCG-3´, reverse 5´-CAGCTTGAGGGTTTGCTACA-3´; *CD209*, forward 5´-GCAGTCTTCCAGAAGTAACCGC-3´, reverse 5´- GCTCTCCTCTGTTCCAATACTGC-3´; *SIRPα*, forward 5´- AGCACTAAGCAACATCTCGCTGTGGACG-3´, reverse 5´- CAAACTGTTAAACCTCAGACTTCACAAGACCC-3´; *TREM2*, forward 5´- TCTGAGAGCTTCGAGGATGC-3´, reverse 5´- GGGGATTTCTCCTTCCAAGA-3´; *TLR-2*, forward 5´- ATCCTCCAATCAGGCTTCTCT-3´, reverse 5´- GGACAGGTCAAGGCTTTTTACA-3´; *TLR-4*, forward 5´- GAGCCTTTTCTGGACTATCAAG-3´, reverse 5´- TCCAATGGGGAAGTTCTCTAG-3´; *CD206*, forward 5´- GCCCGGAGTCAGATCACACA-3´, reverse 5´- AGTGGCTCAACCCGATATGACAG-3´; *CR3*, forward 5´- AATGATGCTTACTTGGGTTATGC-3´, reverse 5´-GCCTGAACATCGCTACCAG-3´; *CD68,* forward 5´-CTTCTCTCATTCCCCTATGGACA-3´, reverse 5´-　　GAAGGACACATTGTACTCCACC-3´.

**Western blotting**

To ensure proper purification of synaptosomes, we performed western blotting for synaptosomal and neuronal proteins. The western blotting procedure was performed using the Simple Western™ system (ProteinSimple, Santa Clara, CA, USA), according to the manufacturer’s protocol. Briefly, pre-made plates with protein specimens, primary antibodies to β-Actin, Synaptophysin (Sigma-Aldrich, #028K4826 and #MAB5258), PSD95, HDAC-2 (Cell signaling technology, Massachusetts, USA, #2507S and #5113S), and NR2B (Merck-Millipore, Damstadt, Germany, #06-600), and secondary antibodies were loaded and run on Wes™. Each sample was analyzed individually in a capillary without interference from adjacent samples. The digital image was analyzed using Compass software, and quantified data of the detected proteins were reported as molecular weight, signal/peak intensity, etc. Quantified area data was used to compare target protein amounts. The results were displayed as a pseudo-blotting pattern.

**Overexpression experiment of *CD209* in M-CSF induced macrophages**

Overexpression of *CD209* was conducted by transfecting plasmid vector using Xfect^TM^ transfection reagent (Takara Bio Inc., Shiga, Japan) according to the manufacturer’s protocol on day3 of culture. Positive vector (pRP[Exp]-Hygro-EF1A>hCD209 [NM_021155.4]) and empty vector (pRP[Exp]-Hygro-EF1A>ORF_Stuffer) were designed (VectorBuilder Japan, Yokohama, Japan). Differentiated macrophages were collected on day six using cell scrapers for phagocytosis assays and qRT-PCR.

**Single cell ATAC sequencing**

**Participants and clinical assessments used in the single-cell multi-omics analysis of PBMCs**

Forty-three individuals with ASD (mean age: 28.9 ± 5.5 years, 11 females) and 18 TD individuals (mean age: 30.1 ± 8.2 years, four females) of Japanese ethnicity were enrolled. All participants were born and had been living in Japan. The participation criteria and the assessment of clinical features were the same as those described in the MATERIALS AND METHODS.

**Sequencing**

To perform single-cell multi-omics analysis of cryopreserved PBMCs, library construction and sequencing were performed following the Chromium Next GEM Single Cell Multiome ATAC + Gene Expression protocol (10x Genomics, CG000338).

**Data analysis of single-cell data**

Sequenced reads from the gene expression (GEX) and DNA accessibility (ATAC) droplet libraries of the single nucleus ATAC and single nucleus RNA assay were processed to generate feature-barcode count matrices of the GEX and ATAC data using the 10x Genomics Cell Ranger ARC pipeline. The reads were subsequently aligned to the pre-built human reference genome GRCh38 provided by 10x Genomics. For each sample, the single cell GEX data and ATAC data were processed with Seurat (version 5.1.0) [1] and Signac (version 1.14.0) [2].

For quality control of GEX data, cells with a high or low number of unique feature counts (top 1% and bottom 1%), as well as those with a high mitochondrial gene rate (top 2%), were filtered out. For quality control of ATAC data, cells meeting the thresholds 3000 < peak_region_fragments < 20000, pct_reads_in_peaks > 15, nucleosome_signal < 2, TSS.enrichment > 2.5 were retained for analysis.

The cell types of PBMC samples were further annotated using the Azimuth PBMC reference, generated from the single-cell transcriptome data and CITE-seq data [1]. Five hundred monocytes from each sample were randomly sampled to equalize each sample's contribution to the downstream analysis. RNA counts for GEX data were then normalized using the SCTransform function. ATAC peaks were called with MACS2 (version 2.2.9.1) [3], using the CallPeaks function in Signac, and were normalized using the term frequency-inverse document frequency (TF-IDF) transformation.

The pseudo-bulk DNA accessibilities of the TD and ASD groups were visualized using the CoveragePlot function. The 500 upstream base pairs and 1000 downstream base pairs of the genomic coding region of the gene to be analyzed were visualized. Differentially expressed genes and differentially accessible regions between the TD and ASD groups were then calculated with the FindMarkers function, and a Wilcoxon Rank Sum test with Bonferroni correction was used for statistical testing.

**Supplementary Figure legends**

**Supplementary Fig. S1**

**Western blotting of synaptosomal and neuronal proteins.**

(A) Western blotting results for SYP, PSD95, NR2B and HDAC-2 are shown as digital images (pseudo-blotting patterns). Beta-actin was used as an internal control, and molecular weight is shown in the leftmost lane. (B) Protein amounts are normalized to β-actin and shown as relative data to neuronal proteins analyzed by Compass software from digital images. The synaptic proteins SYP, PSD95 and NR2B are enriched, while the nuclear protein HDAC-2 is excluded from the synaptosomal proteins. (C, D) Gene expression of (C) *IL-1α* and (D) *IL-10* to characterize GM-CSF MΦ and M-CSF MΦ on day 6 of macrophage differentiation. (E) *CD68* gene expression in M-CSF MΦ during six days differentiation *in vitro*.

**Supplementary Fig. S2**

**Quantitative RT-PCR analysis of TD-M-CSF MΦ in the knockdown experiment.**

(A, B) Gene expression of (A) *IL-1α* and (B) *IL-10* to confirm that siRNA-transfected or sham-treated macrophages maintain the polarization of M-CSF MΦ.

**Supplementary Fig. S3**

**Pseudo-bulk DNA accessibility tracks of the selected gene locus for the TD and ASD groups.**

(A) Visualization of the pseudo-bulk DNA accessibility tracks of the CD209 locus for the TD and ASD groups. Peaks were called with MACS2. (B) The summary of calculating the differentially accessible regions. At all peaks, there were no significant accessibility differences in the DNA accessible regions between the TD and ASD groups.

**Supplementary Fig. S4**

**Overexpression experiment of CD209 in ASD-M-CSF MΦ.**

M-CSF MΦ from four individuals with ASD (one was newly recruited for this analysis) were analyzed. (A) Gene expression of CD209. Wilcoxon matched-pairs signed rank test. (B) Results of the phagocytosis assay. The alteration in phagocytosis capacity exhibited an individual-specific pattern. Paired t test.

**Supplementary Movies 1−8**

Phagocytosis of synaptosomes by GM-CSF MΦ (Movies 1 and 2) and by M-CSF MΦ (Movies 3 and 4) of TD19, and by GM-CSF MΦ (Movies 5 and 6) and by M-CSF MΦ (Movies 7 and 8) of ASD21. Images are acquired every 9 min. Ph, phase contrast; TxsRed, Texas Red Filter.

**Supplementary Movies 9−14**

Phagocytosis of synaptosomes by CD209-siRNA-transfected (Movies 9 and 10), non-targeting-siRNA-transfected (Movies 11 and 12) or sham-treated (Movies 13 and 14) M-CSF MΦ of TD10. Ph, phase contrast; TxsRed, Texas Red Filter.

**Supplementary References**

1. Hao Y, Hao S, Andersen-Nissen E, Mauck WM, 3rd, Zheng S, Butler A et al. Integrated analysis of multimodal single-cell data. *Cell* 2021; **184**: 3573–3587.e3529.

2. Stuart T, Srivastava A, Madad S, Lareau CA, Satija R. Single-cell chromatin state analysis with Signac. *Nature Methods* 2021; **18**: 1333–1341.

3. Feng J, Liu T, Qin B, Zhang Y, Liu XS. Identifying ChIP-seq enrichment using MACS. *Nature Protocols* 2012; **7**: 1728–1740.
