# Supplementary figures and images for: Impaired synaptosome phagocytosis in macrophages of individuals with autism spectrum disorder
Source: Mol Psychiatry. 2025 Apr 4;30(8):3837–45. doi: 10.1038/s41380-025-03002-3 (PMC12240830; doi:10.1038/s41380-025-03002-3)

**A**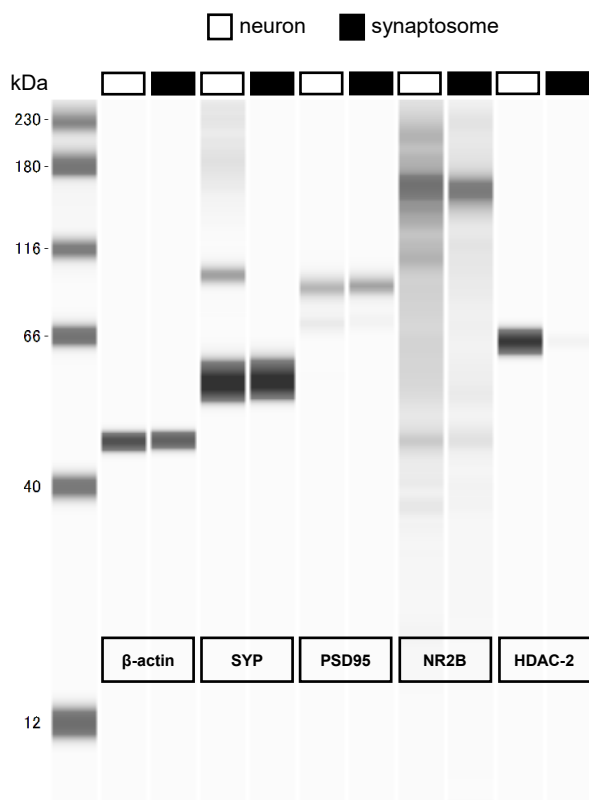**B**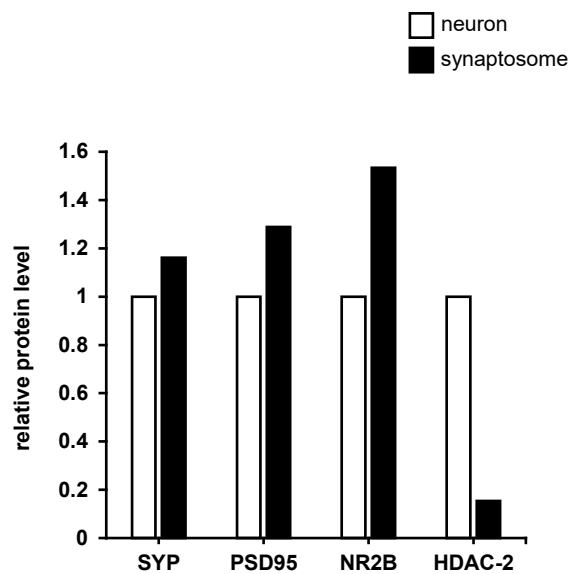**C**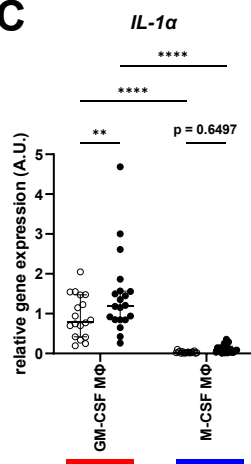**D**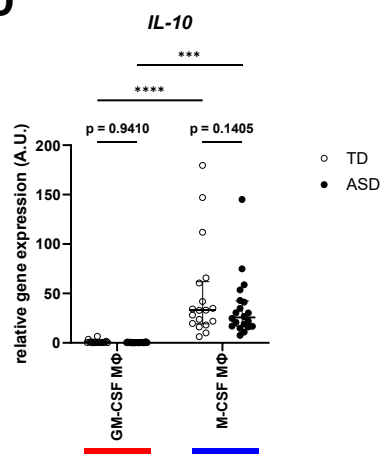**E**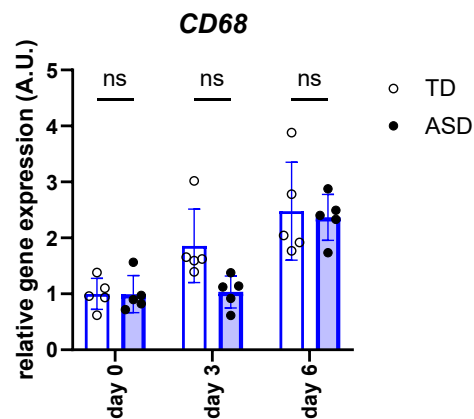

Supplement: Supplementary file 2 — Supplementary Figure 1 [file 41380_2025_3002_MOESM2_ESM.pdf]

**A**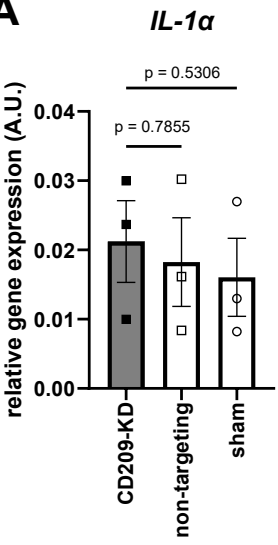**B**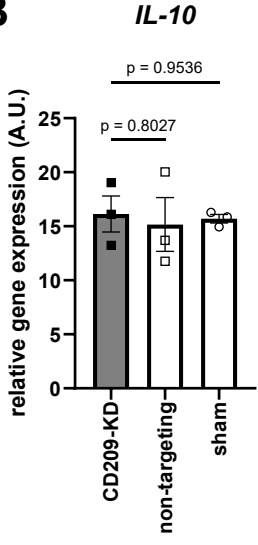**C**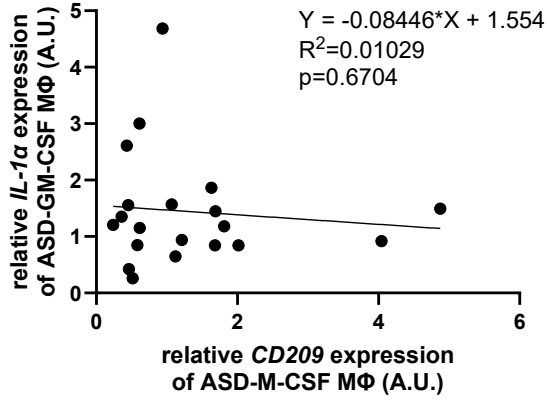

Supplement: Supplementary file 3 — Supplementary Figure 2 [file 41380_2025_3002_MOESM3_ESM.pdf]

A

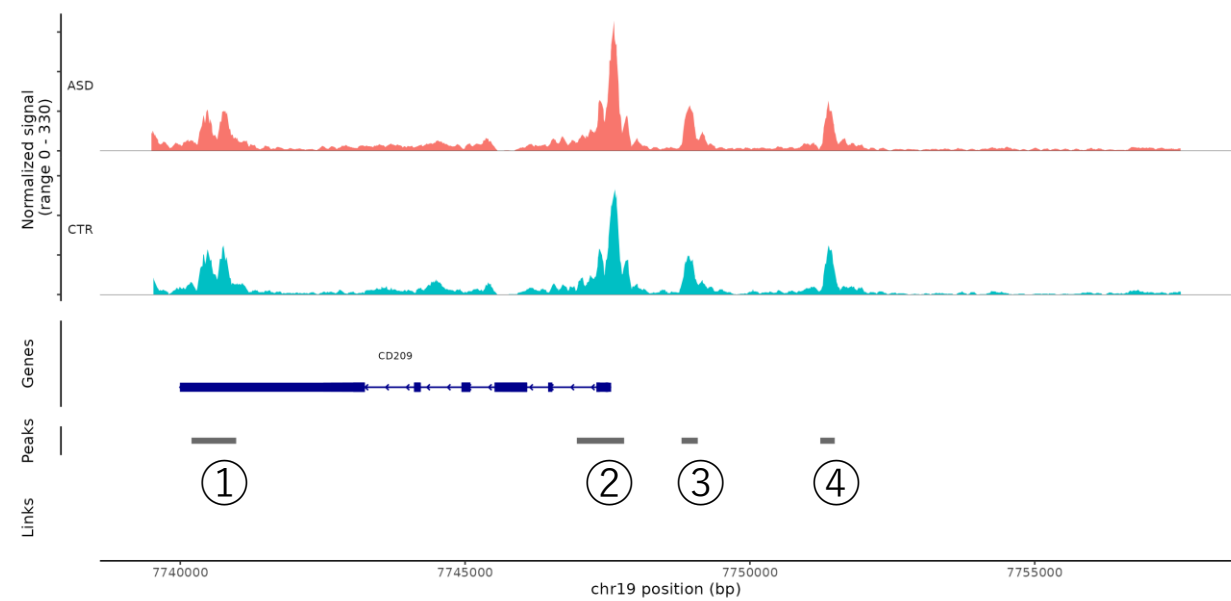

B

|   | peak_region           | p_val       | avg_log2FC   | pct.1 | pct.2 | p_val_adj |
|---|-----------------------|-------------|--------------|-------|-------|-----------|
| ① | chr19-7740198-7740982 | 0.130735998 | -0.140482354 | 0.029 | 0.032 | 1         |
| ② | chr19-7746962-7747792 | 0.204473977 | 0.116083903  | 0.055 | 0.052 | 1         |
| ③ | chr19-7748800-7749085 | 0.577031726 | 0.100855493  | 0.016 | 0.015 | 1         |
| ④ | chr19-7751236-7751489 | 0.917557377 | 0.034029585  | 0.015 | 0.015 | 1         |

Supplement: Supplementary file 4 — Supplementary Figure 3 [file 41380_2025_3002_MOESM4_ESM.pdf]

**A**

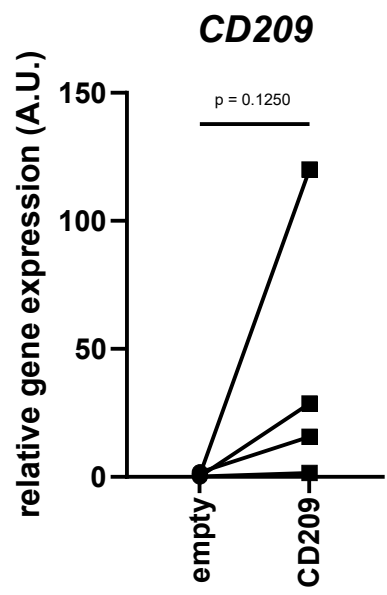

**B**

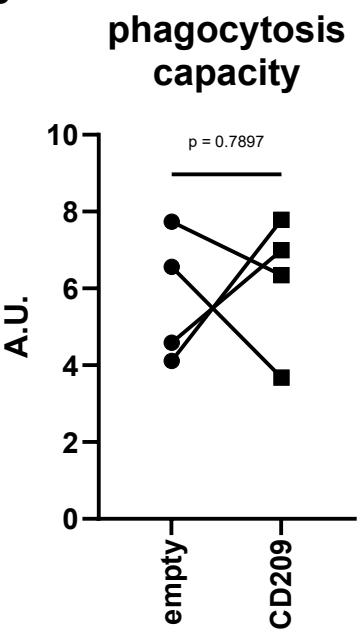

Supplement: Supplementary file 5 — Supplementary Figure 4 [file 41380_2025_3002_MOESM5_ESM.pdf]
